# C‐terminal regulatory domain of the ε subunit of F_o_F_1_ ATP synthase enhances the ATP‐dependent H^+^ pumping that is involved in the maintenance of cellular membrane potential in *Bacillus subtilis*

**DOI:** 10.1002/mbo3.815

**Published:** 2019-02-27

**Authors:** Genki Akanuma, Tomoaki Tagana, Maho Sawada, Shota Suzuki, Tomohiro Shimada, Kan Tanaka, Fujio Kawamura, Yasuyuki Kato‐Yamada

**Affiliations:** ^1^ Department of Life Science, College of Science Rikkyo University Toshima‐ku, Tokyo Japan; ^2^ Research Center for Life Science, College of Science Rikkyo University Toshima‐ku, Tokyo Japan; ^3^ Laboratory for Chemistry and Life Science, Institute of Innovative Science Tokyo Institute of Technology Yokohama, Midori‐ku Japan

**Keywords:** *Bacillus subtilis*, F_o_F_1_‐ATPase, Ribosome, synthases, ε subunit

## Abstract

The ε subunit of F_o_F_1_‐ATPase/synthase (F_o_F_1_) plays a crucial role in regulating F_o_F_1 _activity. To understand the physiological significance of the ε subunit‐mediated regulation of F_o_F_1 _in *Bacillus subtilis*, we constructed and characterized a mutant harboring a deletion in the C‐terminal regulatory domain of the ε subunit (ε^∆C^). Analyses using inverted membrane vesicles revealed that the ε^∆C^ mutation decreased ATPase activity and the ATP‐dependent H^+^‐pumping activity of F_o_F_1_. To enhance the effects of ε^∆C^ mutation, this mutation was introduced into a ∆*rrn8* strain harboring only two of the 10 *rrn* (rRNA) operons (∆*rrn8* ε^∆C^ mutant strain). Interestingly, growth of the ∆*rrn8* ε^∆C^ mutant stalled at late‐exponential phase. During the stalled growth phase, the membrane potential of the ∆*rrn8* ε^∆C^ mutant cells was significantly reduced, which led to a decrease in the cellular level of 70S ribosomes. The growth stalling was suppressed by adding glucose into the culture medium. Our findings suggest that the C‐terminal region of the ε subunit is important for alleviating the temporal reduction in the membrane potential, by enhancing the ATP‐dependent H^+^‐pumping activity of F_o_F_1_.

## 1 INTRODUCTION

F_o_F_1_‐ATPase/synthases (F_o_F_1_) are ubiquitous membrane enzymes that catalyze ATP synthesis from ADP and inorganic phosphate coupled with the H^+^ flow driven by a proton motive force (PMF) that is generated across the bacterial plasma membrane and mitochondrial inner membrane by the respiratory complexes, and across the chloroplast thylakoid membrane by the primary photosynthetic proteins. F_o_F_1_ consists of a water‐soluble F_1_ subcomplex (F_1_‐ATPase) connected to a membrane‐embedded F_o_ subcomplex (Boyer, 1997; Capaldi & Aggeler, 2002; Senior, Nadanaciva, & Weber, 2002; Yoshida, Kobayashi et al., 2001; Yoshida, Muneyuki, & Hisabori, 2001). F_1_‐ATPase is comprised of α_3_, β_3_, γ, δ, and ε subunits. Hydrolysis of an ATP molecule at the catalytic site on the β subunit leads to a discrete 120° rotation of the γε subunits relative to the α_3_β_3_δ (Noji, Yasuda, Yoshida, & Kinosita, 1997). In F_o_F_1_, the coupling of ATP synthesis/hydrolysis and H^+^ flow is achieved by the mechanical rotation of the γε subunits of F_1_ and the ring of c subunits of F_o_, which is responsible for H^+^ translocation. Thus, the primary role of F_0_F_1_ is believed to be the conversion of the PMF into the chemical energy of the phosphoanhydride bond of ATP. On the other hand, the reverse process, ATP‐driven H^+^ pumping, is important for maintaining the cytoplasmic pH in many bacterial species (Kobayashi, Suzuki, & Unemoto, 1986; Liu, Tang, Lin, & Xu, 2015; Shibata, Ehara, Tomura, Igarashi, & Kobayashi, 1992).

The ATP hydrolysis activity of F_1_‐ATPase, which consumes the chemical energy stored in the phosphoanhydride bond of ATP, must be tightly regulated to maintain the physiological function of the cells. One of the universal mechanisms of regulation of F_1_‐ATPase, which affects all known ATP synthases, is inhibition by MgADP (Vasilyeva, Minkov, Fitin, & Vinogradov, 1982; Zhou, Xue, Du, Melese, & Boyer, 1988). Thus, when MgADP, a product of ATP hydrolysis, binds tightly at the catalytic site(s), the F_1_‐ATPase stalls (Feldman & Boyer, 1985; Smith, Rosen, & Boyer, 1983). Although this mechanism of MgADP‐mediated inhibition of ATPases is universal, the extent of inhibition varies considerably. We have found that the ATP hydrolysis activity of *Bacillus subtilis* F_1_‐ATPase (BF_1_), as compared to other F_1_‐ATPases, is highly suppressed by the MgADP (Ishikawa & Kato‐Yamada, 2014; Mizumoto et al., 2013). In BF_1_, the initial ATPase activity, which is not affected by MgADP, rapidly decreases to few percent of the original activity in the steady state as a result of the MgADP inhibition (Ishikawa & Kato‐Yamada, 2014; Mizumoto et al., 2013), whereas the ATPase activity in other ATPases falls only by half to about one‐third of their original activities (Hirono‐Hara et al., 2001; Vasilyeva et al., 1982).

The other well‐known regulatory mechanism of F_1_‐ATPase involves the ε subunit. In general, the ε subunit inhibits ATP hydrolysis activity, although it is believed to contribute to the regulation of ATP synthase (Kato et al., 1997; Laget & Smith, 1979; Ort & Oxborough, 1992; Smith, Sternweis, & Heppel, 1975). The regulatory function of the ε subunit has been shown to depend on its C‐terminal domain (CTD) (Kato‐Yamada et al., 1999; Keis, Stocker, Dimroth, & Cook, 2006; Kuki, Noumi, Maeda, Amemura, & Futai, 1988; Xiong, Zhang, & Vik, 1998), which can adopt two different conformations (Rodgers & Wilce, 2000; Uhlin, Cox, & Guss, 1997; Wilkens & Capaldi, 1998; Yagi et al., 2007). When the CTD of the ε subunit adopts an extended conformation, it reaches into the cavity of the α_3_β_3_ ring and inhibits the ATPase activity (Feniouk, Kato‐Yamada, Yoshida, & Suzuki, 2010; Iino et al., 2005; Rodgers & Wilce, 2000; Tsunoda et al., 2001). However, in the folded‐state conformation, the CTD of the ε subunit is dislodged from the α_3_β_3_ ring, and as a result it does not inhibit the ATPase activity (Kato, Yoshida, & Kato‐Yamada, 2007).

The mechanism of ε subunit‐mediated regulation of F_1_‐ATPase activity, as discussed above, has been delineated by biochemical and crystallographic studies. However, the physiological importance of the ε subunit‐mediated regulation of the F_o_F_1_‐ATPase/synthase activity has received little attention. To address this issue, an *E. coli *mutant harboring a deletion in the CTD of the ε subunit (deletion of residues 94 to 139) was constructed earlier and this deletion mutant was characterized to determine its phenotype (Taniguchi, Suzuki, Berney, Yoshida, & Cook, 2011). The ATPase and the ATP‐dependent H^+^‐pumping activities of F_o_F_1_ (EF_o_F_1_) from this mutant *E. coli* increased due to the absence of ε subunit‐mediated inhibition of activity; these cells exhibited decreased growth rate and lower cell survival in low‐salt medium (Taniguchi et al., 2011). It was also reported that truncation of five residues in the C‐terminal region of the ε subunit reduced the ATP synthesis activity of EF_o_F_1_, resulting in decreased growth rate (Shah & Duncan, 2015). Despite these studies, physiological importance of the ε subunit in the regulation of F_o_F_1_ still remained elusive.

Although several studies have characterized the properties of *B*. *subtilis* F_o_F_1_ (BF_o_F_1_) over the past decades (Hicks, Cohen, & Krulwich, 1994; Hicks & Krulwich, 1987; Santana et al., 1994), the physiological importance of the regulation of BF_o_F_1_ by the ε subunit has not been explored previously. Interestingly, in a recent study using purified BF_1_ we demonstrated that rather than inhibiting BF_1_, the ε subunit activates it by removing the MgADP inhibition (Mizumoto et al., 2013). In the present study, to elucidate the function of the ε subunit in the BF_o_F_1 _holoenzyme and to determine the physiological significance of the regulation of BF_o_F_1 _by the ε subunit, we constructed and characterized a *B. subtilis *deletion mutant (ε^∆C^) strain harboring an ε subunit lacking the CTD region (Lys90–Lys132). Biochemical analyses of inverted membrane vesicles (IMV) prepared from this deletion mutant strain revealed that the CTD of the ε subunit was involved in activating both the ATPase activity and the ATP‐dependent H^+^‐pumping activity of BF_o_F_1_. Furthermore, phenotypic analysis suggested that the CTD of the ε subunit plays an important role in the maintenance of cellular membrane potential. Based on these findings, we also discussed the importance of the regulation of BF_o_F_1 _mediated by the ε subunit.

## 2 MATERIALS AND METHODS

### 2.1 Media and culture conditions

Unless mentioned otherwise, LB [1% Tryptone (Difco), 0.5% yeast extract (Difco), 0.5% NaCl] and LB agar plates were used for growing *B. subtilis *strains. In general, colonies grown on LB agar plates for about 16 hr at 28°C (at 37°C for ∆*rrn8* cells) were inoculated into the appropriate medium, and then grown at 37°C with shaking. Culture conditions and media for preparing competent cells of *B. subtilis* have been described previously (Ashikaga, Nanamiya, Ohashi, & Kawamura, 2000). The composition of the minimal medium, which was based on Spizizen's minimal medium (Anagnostopoulos & Spizizen, 1961), was as follows: 6 g of KH_2_PO_4_, 14 g of K_2_HPO_4_, 2 g of (NH_4_)_2_SO_4_, 1 g of trisodium citrate 2H_2_O per liter of distilled water. When required, 0.1% glucose, 0.1% succinate, and 0.5% casamino acid were added to the minimal medium. Because the *B. subtilis* growth in minimal media with succinate exhibit long lag phase, cells grown to exponential phase (optical density at 600 nm of 0.1) were reinoculated into a minimal medium with succinate, and incubate at 37°C with shaking. When required, 5 µg/ml chloramphenicol, 100 µmol/L CCCP, 1 mmol/L IPTG, 5 µg/ml kanamycin, and appropriate concentrations of glucose were added to the media. Growth curves of *B. subtilis* cells were generated by automatically measuring the OD_660_ value of each culture every 5 min using a TVS062CA incubator (ADVANTEC).

### 2.2 Bacterial strains and plasmids

All of the *B*.* subtilis* strains used in this study were isogenic with *B. subtilis* strain 168 *trpC2.* To insert the chloramphenicol resistance (*cat*) gene 15 bp‐downstream of *atpC*, which codes for the ε subunit of BF_o_F_1_, oligonucleotide primers were used to amplify the regions upstream (atpC+catUF and atpC+catUR) and downstream (atpC+catDF and atpC+catDR) of the insertion site, respectively (see Appendix Table A1 for the nucleotide sequences of all primers used in this study). Next, the *cat *gene from the plasmid pCBB31 (Imamura et al., 2004) was amplified by PCR using the primers atpC+catCF and atpC+catCR. Another PCR reaction, in which all three amplified fragments were added as the DNA template, was carried out using the primers atpC+catUF and atpC+catDR. The resulting fragment was used to transform the strain 168 (*trpC2*), giving rise to a chloramphenicol‐resistant transformant, which was designated as WT ε^wt^ (*atpC* ^+^‐*cat trpC2*). To introduce the A to T nucleotide substitution in the Lys90 codon (AAA) to generate an Ochre stop codon (TAA) and the truncation after codon 90 in the *atpC *gene, we amplified the regions upstream (atpC+catUF and atpC∆CcatR) and downstream (atpC∆CcatF and atpC+catDR) of the mutation site of *atpC* using appropriate primer pairs and chromosomal DNA from the WT ε^wt^ strain as the template. Another PCR reaction, in which both of these amplified fragments were added as the DNA template, was performed using the primers atpC+catUF and atpC+catDR. Strain 168 (*trpC2*) was transformed with the resulting fragment, and chloramphenicol‐resistant transformants, corresponding to the mutant WT ε^∆C^ (*atpC* ^∆C^‐*cat trpC2*), were selected. Furthermore, to introduce the ε^∆C^ mutation into the ∆*rrn8 rrnB* ^+^ *rrnD* ^+^ strain (*trpC2 *∆*rrnHG1 *∆*rrnO1 *∆*rrnE1 *∆*rrnA1 *∆*rrnI2 *∆*rrnW2 *∆*rrnJ1 *∆*ICEBs1*) (Yano et al., 2013), a DNA fragment containing the ε^∆C^ mutation was amplified using primers atpCtfF and atpCtfR and chromosomal DNA from the WT ε^∆C^ (*atpC* ^∆C^‐*cat trpC2*) mutant as the template. The resulting DNA fragment was used to transform the ∆*rrn8 rrnB* ^+^ *rrnD* ^+^ strain, giving rise to a chloramphenicol‐resistant transformant, which was designated as the ∆*rrn8* ε^∆C^ (*trpC2 atpC* ^∆C^‐*cat* ∆*rrnHG1 *∆*rrnO1 *∆*rrnE1 *∆*rrnA1 *∆*rrnI2 *∆*rrnW2 *∆*rrnJ1 *∆*ICEBs1*) strain. The corresponding strain harboring the wild‐type ε^wt^ subunit, strain ∆*rrn8* ε^wt^ (*trpC2 atpC* ^+^‐*cat* ∆*rrnHG1 *∆*rrnO1 *∆*rrnE1 *∆*rrnA1 *∆*rrnI2 *∆*rrnW2 *∆*rrnJ1 *∆*ICEBs1*), was constructed by transforming the ∆*rrn8 rrnB* ^+^ *rrnD* ^+^ strain using a DNA fragment that was amplified by PCR using atpCtfF and atpCtfR as primers and chromosomal DNA from the WT ε^wt^ strain as the template. Correct insertion of the *cat* gene and deletion of the 3’‐region of *atpC *were verified by PCR and DNA sequence analysis. The plasmid pDGatpC, which carries an *atpC* gene controlled by an IPTG‐inducible Pspac promoter, was constructed as follows. The pDG148 (Joseph, Fantino, Herbaud, & Denizot, 2001) and *atpC* gene were amplified using the primers pDGinfF and pDGinfR, and pDGatpCF and pDGatpCR, respectively. The resulting fragments were ligated using In‐Fusion HD Cloning System (TaKaRa). Strains WT ε^∆C^ and ∆*rrn8* ε^∆C^ were transformed with the resulting plasmid, and kanamycin‐resistant transformants were selected.

### 2.3 Preparation of inverted membrane vesicles

*Bacillus subtilis* cells were grown in 400 ml LB medium in a 2 L Erlenmeyer flask at 37°C with shaking (180 rpm) and harvested 5 hr after inoculation. Approximately 100 mg wet weight of cells were first resuspended in 1 ml of MOPS buffer (50 mmol/L MOPS‐KOH pH 7.0, 100 mmol/L K_2_SO_4_, 5 mmol/L MgSO_4_, 10% glycerol) and then treated with 0.1 mg/ml lysozyme at 37°C for 30 min. Lysozyme‐treated cells were disrupted by sonication (TOMY UD‐201; Output 2, Duty 40, 2 min), and the cell debris was removed by centrifugation at 4ºC for 10 min at 20,000× *g*. The cell lysate was centrifuged at 4ºC for 30 min at 200,000× *g*, and the resulting pellet was resuspended in 1 ml of MOPS buffer. After repeating this centrifugation step, the pellet containing inverted membrane vesicles (IMV) was resuspended in 100 µl of MOPS buffer and stored at −80ºC until use.

### 2.4 Measurement of ATP synthesis activity in IMV

The ATP synthesis in the inverted membrane vesicles was measured using a luciferin‐luciferase based fluorescence assay method described previously (Suzuki, Ozaki, Sone, Feniouk, & Yoshida, 2007) and a FP‐6500 fluorescence spectrometer (Jasco). The reaction was carried out at 25ºC in MOPS‐KPi buffer (50 mmol/L MOPS‐KOH pH 7.0, 100 mmol/L K_2_SO_4_, 5 mmol/L MgSO_4_, 10 mmol/L KPi, 0.5 mmol/L ADP, 10 µmol/L diadenosine pentaphosphate). At 1 min after incubation of MOPS‐KPi buffer at 25ºC, 100 µl of the CLSII solution containing luciferin/luciferase (ATP Bioluminescence Assay Kit CLSII; Roche) was added and then 5 min later, IMV (containing 60 µg of membrane proteins) was added. At 1 min after the addition of IMV, the reaction was initiated by the addition of 1.8 mmol/L succinate to generate an electrochemical potential gradient of H^+^ across the membrane. The solution was stirred continuously during the measurement. The ATP synthesis activity was monitored in real time by measuring the intensity of light emitted from the luciferase reaction at 560 nm. Reaction rates were determined at various times (1–4 min). The amount of ATP produced was calibrated with a defined amount of ATP at the end of the measurement. One unit of activity was defined as the amount that synthesized 1 µmol of ATP per min.

### 2.5 Measurement of ATP hydrolysis activity in IMV

ATPase activity was measured using an NADH‐coupled ATP regenerating system at 25ºC using a modified protocol of a previously described method (Kadoya, Kato, Watanabe, & Kato‐Yamada, 2011). Briefly, the assay mixture (1.5 ml), consisting of 50 mmol/L MOPS‐KOH pH 7.0, 100 mmol/L K_2_SO_4_, 5 mmol/L MgSO_4_, 2.5 mmol/L phosphoenolpyruvate, 50 µg/ml lactate dehydrogenase, 50 µg/ml pyruvate kinase, 0.3 mmol/L NADH, 0.1 µmol/L valinomycin, 1 µg/ml carbonyl cyanide p‐trifluoromethoxyphenylhydrazone, 40 mmol/L KCN and 10% glycerol, was transferred to a glass cuvette. At 4.5 min after incubation at 25ºC, membrane vesicles (IMV containing 60 µg of membrane proteins) were added to the assay mixture, and absorbance at 340 nm was measured using a V‐550 spectrophotometer (JASCO) at 1 s intervals. Because the rate of ATP hydrolysis was determined from the rate of NADH oxidation, the rate of consumption of NADH by the respiratory complex was determined during this period (230–290 s after addition of IMV). At 5 min after addition of IMV, 2 mmol/L ATP was added to the assay mixture. The mixture was stirred with a magnetic stirrer for 5 s before and after the addition of IMV and ATP. Finally, the ATPase activity was calculated by subtracting the NADH consumption rate of the respiratory chain from the ATPase reaction rate.

### 2.6 Measurement of H^+^ pumping activity in IMV

H^+^ pumping activity of F_o_F_1_, driven by ATP and that by the respiratory complex energized with succinate, was measured at 25°C by measuring the quenching of fluorescence of 9‐amino‐6‐chloro‐2‐methoxyacridine (ACMA) as described previously with some modifications (Suzuki et al., 2007). A fluorescence spectrometer FP‐6500 (Jasco) was used in the assay to measure the emitted fluorescence. The reaction was initiated by the addition of 2 mmol/L ATP or 5 mmol/L succinate to a quartz cuvette that contained 2 ml of MOPS buffer (50 mmol/L MOPS‐KOH pH 7.0, 100 mmol/L K_2_SO_4_, 5 mmol/L MgSO_4_, 10% glycerol) supplemented with 0.15 µg/ml ACMA, 0.1 µmol/L valinomycin and membrane vesicles containing 50 µg of membrane proteins. At 5 min after the addition of ATP or succinate, 0.5 μg/ml CCCP (carbonyl cyanide m‐chlorophenylhydrazone) was added to dissipate the H^+^ gradient. Fluorescence intensity after the addition of CCCP was set to 1. To measure the fluorescence, the excitation and emission wavelengths, λ_ex _and λ_em_, were set at 410 and 480 nm, respectively, with 5 nm bandwidths.

### 2.7 Measurement of cellular ATP concentration

*Bacillus* *subtilis* cells were grown in 5 ml LB medium in a glass L‐shaped test tube at 37°C with shaking and harvested from 200 µl culture at the indicated times. Simultaneously, viable cells were counted by plating the culture on LB agar plates and then incubating the plates at 37°C until colonies were detected. For the luciferase assay, cells were suspended in luciferase sampling buffer (100 mmol/L Tris‐HCl, pH 7.75; 4 mmol/L ethylenediaminetetraacetic acid) with an equal volume of the harvested culture. Fifty microlitres of cell suspension was mixed with 450 µl of luciferase sampling buffer preheated to 95°C. The mixture was incubated at 95°C for 3 min, and then transferred to ice. The luciferase assay was performed with a Lumat LB9507 luminometer (Berthold) using the ATP Bioluminescence Assay Kit CLS II (Roche) according to the manufacturer's instructions. The ATP content per cell was calculated by dividing the amount of ATP in the cell suspension by the number of viable cells. The cellular ATP concentration was calculated by assuming that a *B. subtilis* cell is a cylinder. To measure the cell size (radius and length) at the indicated times, microscopic images were analyzed by MicrobeJ, an ImageJ plug‐in (Jiang, Brown, Ducret, & Brun, 2014). The mean size of >30 cells was used for the calculation.

### 2.8 Detection of cellular membrane potential

Cells were grown in 5 ml LB medium in a glass L‐shaped test tube at 37°C with shaking and harvested at the indicated times. Cellular membrane potential was detected by using a *Bac*Light™ Bacterial Membrane Potential Kit (Molecular Probes) according to the manufacturer's instructions. Cells were stained with DiOC_2_(3) (3,3’‐diethyloxacarbocyanine iodide) to detect membrane potential and stained with TO‐PRO^®^‐3 (Molecular Probes) to detect cells with damaged membranes. A BD FACS Verse^TM^ flow cytometer (BD Biosciences) was used to detect fluorescence. The stained cells were analyzed using a 488 nm excitation laser and 527/32 nm and 613/18 nm emission filters to detect green and red fluorescence from DiOC_2_(3), respectively. Simultaneously, a 640 nm excitation laser and 660/10 nm emission filter was used to detect the fluorescence from TO‐PRO^®^‐3. The photomultiplier tube (PMT) voltages for green and red fluorescence from DiOC_2_(3) were 261 V and 393 V, respectively. The data, corresponding to cells whose fluorescence intensity of TO‐PRO^®^‐3 were larger than 131 when the PMT voltage was 200 V, were eliminated because membranes of these cells are likely damaged. It should be noted that even at a higher PMT voltage, the population of cells showing high fluorescence intensity of TO‐PRO^®^‐3 was not large, indicating that very few cells used in this assay had damaged membrane.

### 2.9 Measurement of the dissolved oxygen concentration in the culture medium

Cells were inoculated into 100 ml LB medium in a glass T‐shaped connecting tube, and then the dissolved oxygen concentration of the culture was automatically measured every 20 min by using a FIBOX3 oxygen meter (TAITEC) at 37°C with shaking. It was confirmed that the growth curves of all strains in this assay were basically the same as those in small‐scale batch cultures in glass L‐tubes.

### 2.10 Measurement of the glucose concentration in the medium

Cells were grown in 5 ml LB medium in a glass L‐shaped test tube at 37°C with shaking, and the culture supernatant was collected by centrifugation at the indicated times. Proteins that were present in the culture supernatant were removed using a Deproteinizing Sample Preparation Kit (Abcam) according to the manufacturer's instructions. After removing proteins, the glucose concentration in the culture supernatant was measured using a Glucose Assay Kit (Abcam) according to the manufacturer's instructions.

### 2.11 Sucrose density gradient centrifugation

Sucrose density gradient centrifugation was carried out to analyze ribosome profiles. Cells taken from LB plates were used to inoculate 400 ml of LB medium in a 2 L Erlenmeyer flask and were grown at 37°C with shaking (180 rpm). Aliquots of cell culture [volume (ml) × OD_600_ = 100] were removed at the indicated times and cells from each aliquot were harvested by centrifugation. Sucrose density‐gradient centrifugation was performed as described previously (Akanuma et al., 2014). Briefly, an aliquot of each crude cell extract (10 *A*_260_ units each) was layered onto a 10–40% sucrose density gradient, and the gradient was centrifuged at 65,000× *g* in a Hitachi P40ST rotor for 17.5 hr at 4°C. After centrifugation, the gradient was fractionated using a Piston Gradient Fractionator (BioComP) and absorbance profiles were monitored at 254 nm using a Bio‐mini UV Monitor (ATTO).

### 2.12 Western blot analysis

Cells taken from LB plates were used to inoculate 200 ml of LB medium in a 1 L Erlenmeyer flask and were grown at 37°C with shaking (180 rpm). Aliquots of cell culture [volume (ml) × OD_600_ = 5] were removed at the indicated times and cells from each aliquot were harvested by centrifugation. It was confirmed that the growth curves of all of the strains in this assay were basically the same as those in small‐scale batch cultures in glass L‐tubes. Western blot analysis was performed following a method described previously (Nanamiya et al., 2003). Crude cell extracts, each containing 20 µg of total protein, were electrophoresed on a sodium dodecyl sulfate‐polyacrylamide (10–20%) minigel and the separated proteins were transferred to a polyvinylidene difluoride membrane (Millipore Co.). This membrane was then used in the Western blot assay using antisera (1:1,000 dilution) against the β and γ subunits of TF_1_, which were kindly provided by Toshiharu Suzuki and Masasuke Yoshida.

## 3 RESULTS

### 3.1 Deletion of the C‐terminal domain of the ε subunit decreased the ATPase activity and the ATP‐dependent H^+^‐pumping activity of BF_o_F_1_

**Figure 1:**
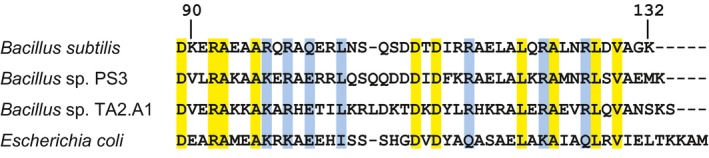
Alignment of the C‐terminal region of the ε subunit. Identical and similar amino acids among four species are highlighted with yellow and blue, respectively. Sources of the amino acid sequences were as follows; *Bacillus subtilis* (Santana et al., 1994), thermophilic *Bacillus *PS3 (Kato‐Yamada, Yoshida, & Hisabori, 2000), *Bacillus* sp. TA2.A1. (Keis, Kaim, Dimroth, & Cook, 2004), and *E. coli* (Walker, Saraste, & Gay, 1984)

**Figure A1:**
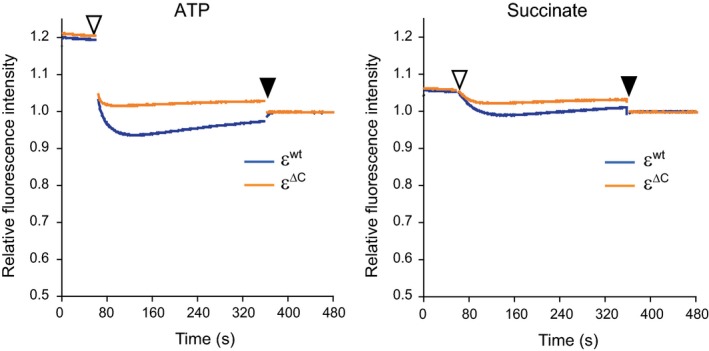
The H^+^‐pumping activities of F_o_F_1_ (left) and respiratory complex (right) in the IMVs at 37ºC. Experiments were carried out as in Figure 2c but at 37ºC

To elucidate the function and the physiological significance of the regulation of BF_o_F_1 _by the ε subunit, we constructed a deletion mutant lacking the CTD of the ε subunit (WT ε^∆C^). In the ε subunit of F_o_F_1_ of thermophilic *Bacillus* PS3 (TF_o_F_1_), which shares 69% identity with the ε subunit of BF_o_F_1_, the C‐terminal helical domain is formed starting from the Val90 residue (Yagi et al., 2007). Sequence alignment analysis revealed that Val90 of the ε subunit of TF_o_F_1_ corresponded to the Lys90 of that of BF_o_F_1_ (Figure 1). Thus, we altered the Lys90 codon (AAA) of the *atpC *gene encoding the ε subunit to an Ochre stop codon (TAA) by nucleotide substitution and truncated the gene after codon 90 to create the desired deletion mutant. The *cat* gene, which encoded chloramphenicol acetyltransferase and was employed as a selectable marker, was also introduced downstream of the mutated *atpC *gene. This insertion of the *cat *gene probably did not affect the expression of other genes, because the *atpC *gene, which is encoded in an operon with the other BF_o_F_1 _subunits, is located at the end of the *atp* operon. In this respect it should be noted that the orientation of the *ywmA *gene, which is located downstream of the *atp* operon, is different from that of *atpC*. However, to ensure that the *cat *gene did not have any effect on the expression of other genes, we created and used a strain harboring the *cat* gene downstream of the intact *atpC *gene (WT ε^wt^) as a control. For biochemical characterization of BF_o_F_1_ containing ε^∆C^, we then prepared inverted membrane vesicles (IMV) from the WT ε^wt^ and WT ε^∆C^ cells. Initially, we tried to measure the activities in these IMVs at 37°C which is the optimal temperature for growth of *B. subtilis*. However, we could not measure the F_o_F_1_ as well as the respiratory complex activity of H^+^‐pumping at 37°C, possibly due to their relatively low activities and/or the H^+^‐permeability of the lysozyme‐treated IMV at this temperature (see Appendix, Figure A1). Therefore, biochemical analyses of IMV were performed at 25°C which is the optimal temperature for H^+^‐pumping activity measurements for both of F_o_F_1_ and the respiratory complex in IMV.

**Figure 2:**
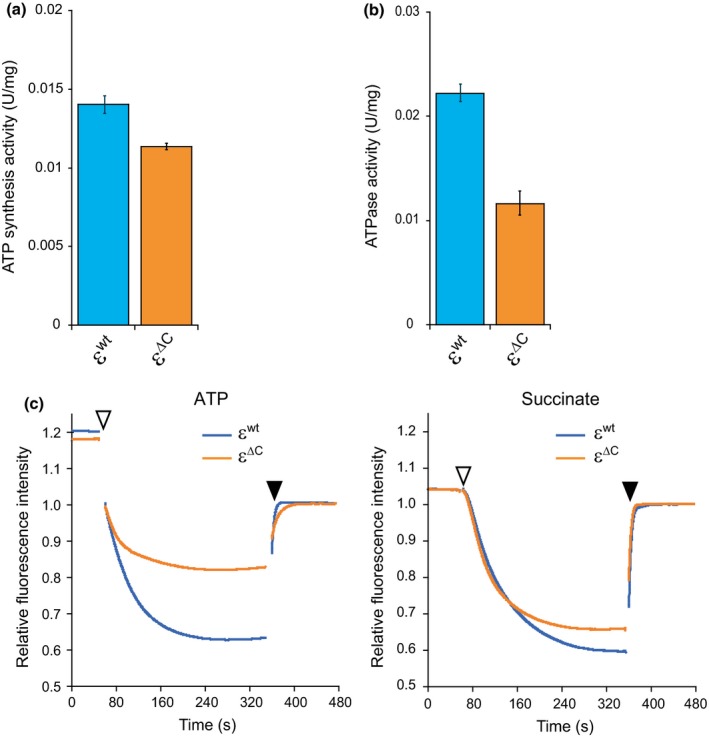
Analysis of enzyme activities of BF_o_F_1_. IMVs prepared from the WT ε^wt^ and WT ε^∆C^ mutant cells were used in the activity assays. (a) ATP synthesis by BF_o_F_1_, driven by the H^+^ electrochemical gradient across the membrane, was measured using a luciferin‐luciferase based assay method as described in the Materials and Methods. The enzyme activity needed to synthesize 1 µmol of ATP per min was defined as 1 unit. Data shown are ATP synthesis activity per mg of membrane protein. In (a) and (b), results shown are averages of three experiments and error bars indicate standard errors. The blue columns and orange columns indicate WT ε^wt^ and WT ε^∆C^, respectively. (b) ATP hydrolysis activity of the F_o_F_1_ was measured using a NADH‐coupled ATP regenerating assay method. The activity that hydrolyzed 1 µmol of ATP per min was defined as 1 unit. Data shown are ATP hydrolysis activity per mg of membrane protein. (c) H^+^‐pumping activities of F_o_F_1_ (left) and respiratory complex (right). Pumping activity was measured by monitoring quenching of fluorescence intensity of ACMA as described in the Materials and Methods. The assay reaction was initiated by adding either 2 mmol/L ATP (left plot) or 5 mmol/L succinate (right plot) at the times indicated by open arrow heads and terminated with the addition of CCCP at the times indicated by closed arrow heads. Fluorescence intensity after the addition of CCCP (at 480 s) was defined as 1. The blue lines and orange lines indicate WT ε^wt^ and WT ε^∆C^, respectively

**Figure A2:**
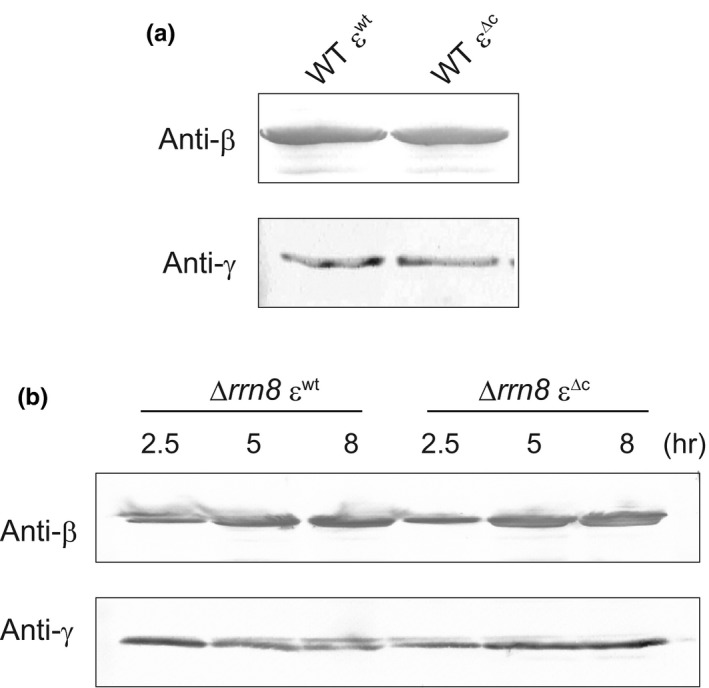
F_o_F_1_ contents in the membrane fraction, as defined by the amounts of β and γ subunits. (a) Contents of b and g subunits in the IMV (20 μg protein per sample), which were prepared from WT ε^wt^ and WT ε^∆C^ cells and used for the analyses to determine the enzymatic activity of the F_o_F_1_, were detected by Western blotting using antisera against the β and γ subunits as described in Experimental procedures. (b) Contents of β and γ subunits in the membrane fraction (20 μg protein per sample), which were prepared from ∆*rrn8* ε^wt^ and ∆*rrn8* ε^∆C^ cells grown in LB medium and harvested at the indicated times, were detected by Western blotting. The membrane fractions were prepared as follows. Cells were resuspended in lysis buffer (20 mmol/L Tris‐HCl pH 8.0, 2 mmol/L phenylmethylsulfonyl fluoride, 0.1 mmol/L dithiothreitol) and disrupted by sonication (TOMY UD‐201; Output 4, Duty 40, 1 min). After removing cell debris by centrifugation at 4ºC for 10 min at 20,000× g, the cell lysate was centrifuged at 4ºC for 30 min at 200,000× g, and the resulting pellet was resuspended in lysis buffer

Next, we measured the ATP‐synthesis activity present in these IMVs using a luciferin‐luciferase based assay. It should be noted that the ε^∆C ^mutation had no effect on the amount of BF_o_F_1_ in the IMV, as was assessed by measuring the amounts of γ and β subunits by western blot analysis (Figure A2a). The rate of ATP synthesis in the IMV of WT ε^∆C^ was about 19% less than that in the IMV of WT ε^wt^ (Figure 2a). The H^+^‐pumping activity of the respiratory complex that produces the PMF also decreased slightly in the IMV of WT ε^∆C^ (Figure 2c, right plot). In this luciferin‐luciferase based assay system that was employed here, BF_o_F_1_ utilized PMF, which was generated by the respiratory complex, for the synthesis of ATP. Assuming that the decrease in the H^+^‐pumping activity of the respiratory complex in the IMV of WT ε^∆C^ affected the PMF, the decrease in the ATP synthesis activity of BF_o_F_1_ due to the ε^∆C^ mutation might be <19%. The F_o_F_1_ not only can synthesize ATP coupled with the H^+^ flow driven by the PMF, it can also reverse the direction of the H^+^ flow by hydrolyzing ATP (Boyer, 1997; Capaldi & Aggeler, 2002; Senior et al., 2002; Yoshida, Kobayashi et al., 2001; Yoshida, Muneyuki et al., 2001). Thus, we next determined the effect of the ε^∆C^ mutation on the ATP hydrolysis activity of BF_o_F_1_. As shown in Figure 2b, the ATPase activity of BF_o_F_1 _containing the ε^∆C^ mutation decreased by 48% relative to that of the intact BF_o_F_1_. This result correlates with our previous finding that the ATP hydrolysis activity of the purified BF_1_‐ATPase lacking the ε subunit was less than that of the BF_1_‐ATPase containing the intact ε subunit (Mizumoto et al., 2013). The H^+^‐pumping activity of BF_o_F_1 _containing the ε^∆C^ mutation is expected to be less than that of the intact BF_o_F_1 _because ATP hydrolysis is coupled to H^+^‐pumping. As expected, the ε^∆C^ mutation decreased the ATP‐dependent H^+^‐pumping activity of BF_o_F_1 _(Figure 2c, left plot). Therefore, it is very likely that the observed reduction in the H^+^‐pumping activity of BF_o_F_1 _containing the ε^∆C^ mutation was caused by the decrease in the ATP hydrolysis activity.

### 3.2 Phenotypic analysis of the ε^∆C^ mutant

**Figure 3:**
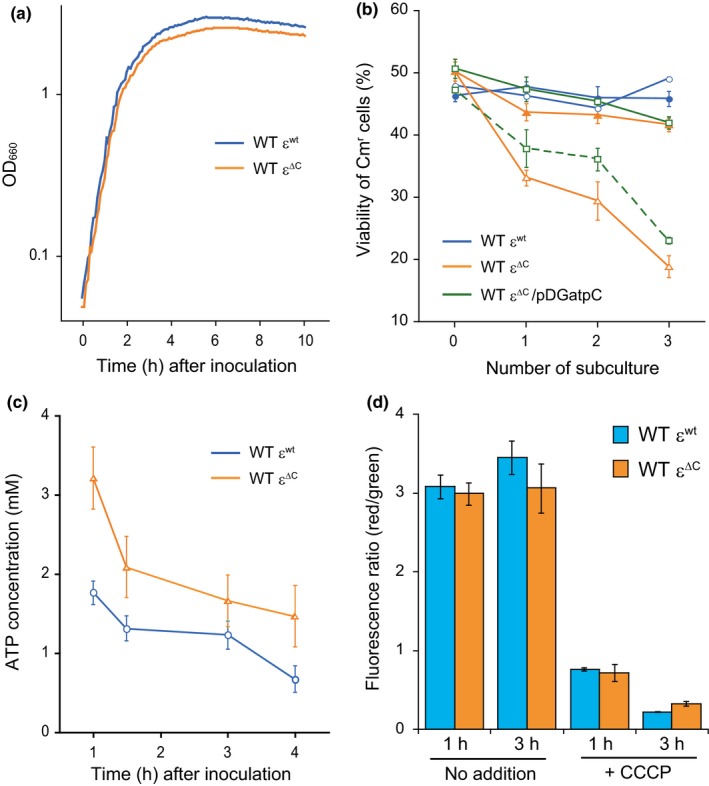
Phenotypic analysis of the WT ε^∆C^ mutant. (a) For growth curves, WT ε^wt^ (blue line) and WT ε^∆C^ mutant (orange line) cells were grown in LB, and OD_660_ value of each culture was measured every 5 min. (b) Mixed cultures of wild‐type and WT ε^wt^ cells or wild‐type and WT ε^∆C^ mutant cells were incubated at 37°C with shaking either in LB medium (open symbols) or in LB medium supplemented with 0.1% glucose (closed symbols). The relative viable counts of WT ε^wt^ cells (blue circles) and WT ε^∆C^ cells (orange triangles) are shown. Mixed cultures of wild‐type/pDG148 (empty vector) cells and WT ε^∆C^/pDGatpC cells, which can be induced an expression of intact ε subunit by addition of IPTG, were incubated at 37°C with shaking in LB medium with (green solid line) or without (green dashed line) IPTG. The relative viable counts of WT ε^∆C^/pDGatpC cells (green squares) are also shown. (c) Cellular concentrations of ATP in the WT ε^wt^ (blue circles) and WT ε^∆C^ (orange triangles) cells. Cells were grown in LB medium and harvested at the indicated times. The concentration of ATP in cells was determined as described in the Materials and Methods. (d) The relative membrane potentials of the WT ε^wt^ (blue columns) and WT ε^∆C^ (orange columns) cells in the presence and absence of the uncoupler CCCP were measured using the *Bac*Light™ Bacterial Membrane Potential Kit (Molecular Probes). DiOC_2_(3), a dye used in this assay, exhibits green fluorescence in all cells, but the fluorescence shifts toward red emission as the dye molecules self‐associate at higher cytosolic concentrations caused by an increase in the membrane potential. Thus, cells that with higher membrane potential show higher fluorescence ratio (red/green). In (b), (c), and (d), results shown are averages of three independent experiments and error bars indicate standard errors.

Biochemical analyses using IMV revealed the effects of the ε^∆C^ mutation on the catalytic activity of BF_o_F_1_. Results obtained from these experiments prompted us to characterize living WT ε^∆C^ mutant cells in order to elucidate the physiological significance of the regulation of BF_o_F_1_ activity mediated by the ε subunit. Although the ε^∆C^ mutation did not affect the growth rate in LB medium at 37°C, the optical density at 660 nm (OD_660_) of the culture of the WT ε^∆C^ mutant cells in the early‐stationary phase was slightly less than that of the WT ε^wt^ cells (Figure 3a). If this difference reflected the influence of the ε^∆C^ mutation on the cell number, it is expected that the WT ε^∆C^ mutant would show a competitive survival disadvantage when co‐cultured with the wild‐type cells. To test this hypothesis, relative survival rates of the wild‐type (parental strain; 168 *trpC2*) and WT ε^∆C^ mutant cells, which were grown in mixed culture and repeatedly subcultured, were determined. In this experiment, WT ε^wt^ and WT ε^∆C^ mutant cells, both of which are chloramphenicol‐resistant, were mixed with the wild‐type cells, which are chloramphenicol‐sensitive. Accordingly, both wild‐type and WT ε^∆C^ mutant cells (or wild‐type and WT ε^wt^ cells) were grown together in LB medium for 8 hr, and then the culture was appropriately diluted and aliquots were plated on LB agar plates with or without chloramphenicol. The culture was appropriately diluted every 24 hr with fresh LB medium, and this subculturing process was repeated three times. It should be noted that the sporulation frequency of these strains would not affect the result, because *B. subtilis* cells hardly form spore in LB medium at 24 hr (<0.01%) (Akanuma et al., 2016). As expected, the relative abundance of WT ε^∆C^ mutant cells decreased compared to that of the wild‐type cells at each subculture, whereas that of WT ε^wt^ cells was maintained (Figure 3b). It was confirmed that this phenotype of WT ε^∆C^ mutant could be suppressed by expression of intact *atpC*, encoding the ε subunit, which is controlled by an IPTG‐inducible Pspac promoter (Figure 3b). These results suggest that the CTD of the ε subunit is important for survival advantage under competitive condition.

We next investigated the effect of ε^∆C^ mutation on the cellular concentration of ATP and also on the membrane potential, because experiments using IMV showed decreased ATP hydrolysis and H^+^‐pumping activities of BF_o_F_1_ containing the ε^∆C^ mutation (Figure 2b,c). Cellular concentrations of ATP in the WT ε^wt^ and WT ε^∆C^ mutant cells, grown in LB medium until early‐stationary phase, were measured. In the ε^∆C^ mutant cells, the ATP concentration was higher than that in ε^wt^ cells at all time points examined (Figure 3c). In particular, the ATP concentration in ε^∆C^ mutant cells was highest at early‐exponential phase (Figure 3c). This result implies that the reduction in the ATPase activity of F_o_F_1 _caused by the ε^∆C^ mutation affects the cellular ATP concentration at least in part, although it was unclear whether the effect was direct or indirect. On the other hand, the ε^∆C^ mutation hardly affected the membrane potential of exponentially growing cells, although the membrane potential of the ε^∆C^ mutant at early‐stationary phase was slightly decreased (Figure 3d). We confirmed that the addition of carbonyl cyanide m‐chlorophenylhydrazone (CCCP; a protonophore), which causes the dissipation of the PMF, clearly abolished the membrane potential in both WT ε^wt^ and WT ε^∆C^ mutant cells (Figure 3d).

### 3.3 Effects of deletion of the C‐terminal domain of the ε subunit in a ∆*rrn8* mutant

**Figure 4:**
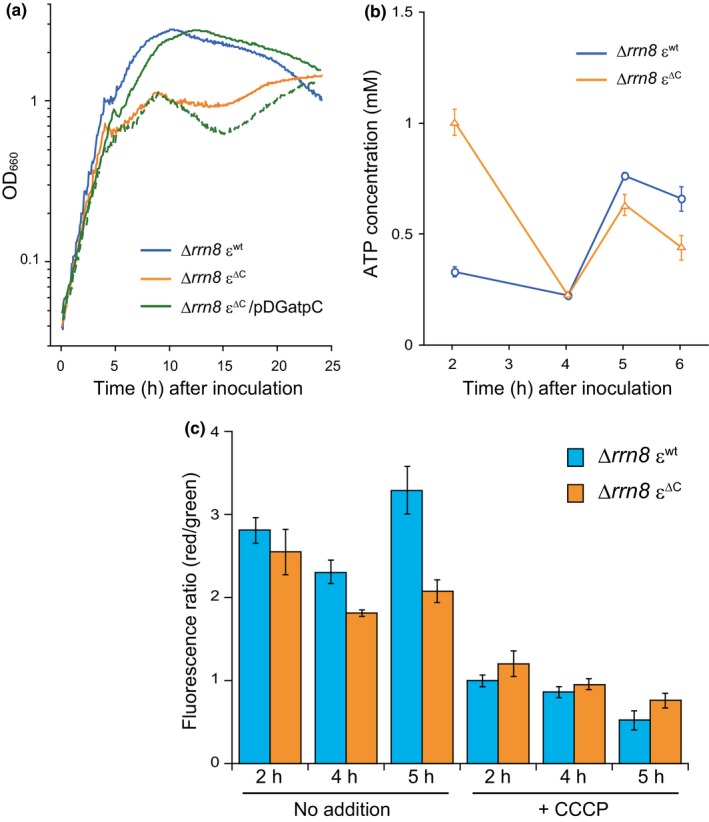
Phenotypic analysis of the ∆*rrn8* ε^∆C^ mutant. (a) For growth curves, ∆*rrn8* ε^wt^ (blue line) and ∆*rrn8* ε^∆C^ mutant (orange line) cells were grown in LB, and ∆*rrn8* ε^∆C^ /pDGatpC cells were grown in LB with (green solid line) or without (green dashed line). OD_660_ of each culture was measured every 5 min. (b) Cellular concentrations of ATP in the ∆*rrn8* ε^wt^ (blue circles) and ∆*rrn8* ε^∆C^ (orange triangles) cells were determined as described above in the legend of Figure 3c. (c) Cells were grown in LB medium and harvested at the indicated times. Relative membrane potential in untreated and CCCP treated cells was measured as described above in the legend of Figure 3d. Cells with higher membrane potentials exhibited higher fluorescence ratios (red/green). In (b) and (c), results shown are averages of three independent experiments and error bars indicate standard errors.

**Figure A3:**
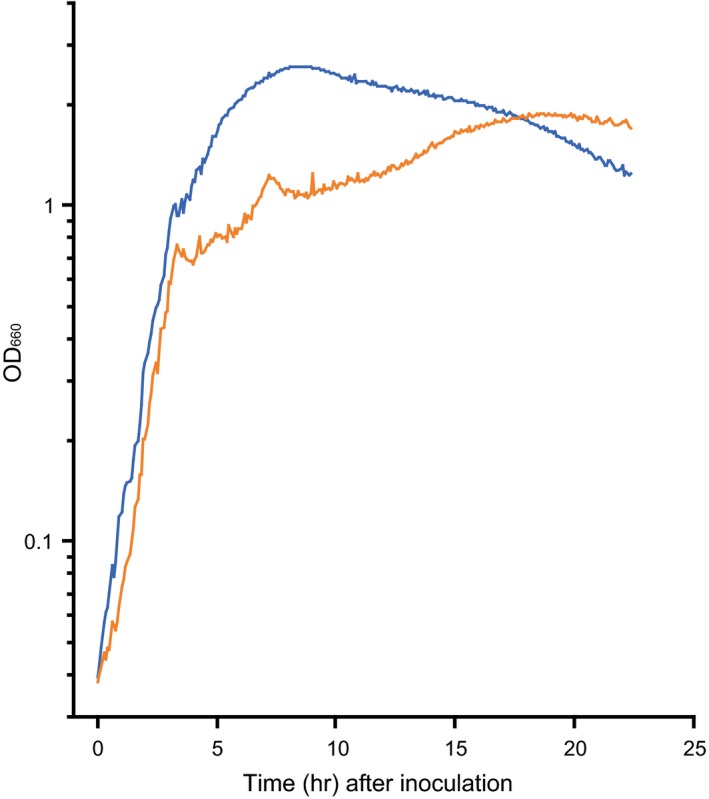
The ∆*rrn8 rrnA* ^+^ *rrnO* ^+^ ε^∆C^ mutant also showed growth‐stalling phenotype. The ∆*rrn8 rrnA* ^+^ *rrnO* ^+^ ε^wt^ (blue line) and ∆*rrn8 rrnA* ^+^ *rrnO* ^+^ ε^∆C^ mutant (orange line) cells were grown in LB, and OD_660_ of each culture was measured every 5 min

To understand why the deletion of the CTD of the ε subunit caused a survival disadvantage to the cells harboring this mutation under competitive condition (Figure 3b), the effects of the ε^∆C^ mutation were explored in adverse genetic backgrounds. As described above, results of various assays performed using IMVs suggested that the CTD of the ε subunit was important for the regulation of ATPase activity as well as the H^+^‐pumping activity of BF_o_F_1_. We predicted that this function of the ε subunit would be valuable for cells when adapting to environmental changes. Some of the well‐known mechanisms by which cells adapt to environmental changes include regulation of gene expression and regulation of enzymatic activities. These two regulatory mechanisms could possibly work in coordination, for example, as was shown in the glycolytic pathway (Fillinger et al., 2000; Yoshida, Kobayashi et al., 2001; Yoshida, Muneyuki et al., 2001), and thereby, would make it possible for the cells to rapidly adapt to environmental changes. Accordingly, any missing link between these two regulatory mechanisms would be expected to delay the adaptation of cells to environmental changes. Therefore, to accentuate the effects of the ε^∆C^ mutation, we introduced this mutation into the ∆*rrn8* cells, in which the regulation of gene expression is delayed as a result of decreased translational activity. Previously, we constructed mutant strains harboring one to nine copies of the rRNA (*rrn*) operon in the *B. subtilis* genome (wild type contains 10 copies) and confirmed that the number of ribosomes is reduced in these mutants, especially in those that harbor only one or two *rrn* operons (Nanamiya et al., 2010; Yano et al., 2013). Because of the frequent appearance of suppressor mutations and difficulty in transforming mutants harboring only one *rrn *operon (Yano et al., 2016, 2013), the ε^∆C^ mutation was introduced into a mutant harboring only the *rrnB *and *rrnD* operons, whose growth rate is particularly slow as compared to the other ∆*rrn8* mutants (Yano et al., 2013), and created the ∆*rrn8 rrnB* ^+^ *rrnD* ^+ ^ε^∆C^ (∆*rrn8* ε^∆C^) mutant strain. When these ∆*rrn8* ε^∆C^ mutant cells were grown in LB medium at 37°C, their growth rate in the exponential phase was almost same as that of the ∆*rrn8* ε^wt^ cells, which had the *cat* gene inserted downstream of the intact *atpC*. However, interestingly, the growth of ∆*rrn8* ε^∆C^ mutant cells began to stall approximately 4 hr after the inoculation (Figure 4a). We confirmed that the growth‐stalling caused by ε^∆C^ mutation was suppressed by expression of intact ε subunit in the ∆*rrn8* ε^∆C^ mutant cells (Figure 4a). It should be noted that a similar growth‐stalling effect was also observed for the ∆*rrn8 rrnA* ^+^ *rrnO* ^+ ^ε^∆C^ mutant cells whose growth rate was slightly faster than that of the ∆*rrn8 rrnB* ^+^ *rrnD* ^+ ^ε^∆C^ (∆*rrn8* ε^∆C^) mutant cells (Figure A3). Although it is possible that this observed phenotype of the ∆*rrn8* ε^∆C^ mutant cells was due to a decrease in the amount of BF_o_F_1_ in the cell membrane, we compared the abundances of β and γ subunits by western blotting, the components of F_1_, in the cell membrane and found that their abundances in both ∆*rrn8* ε^∆C ^and ∆*rrn8* ε^wt^ cells were same (Figure A2b). We observed mutant cells by microscopy to investigate whether the ∆*rrn8* ε^∆C ^mutant exhibit change of cell shape, however, no significant difference of cell shape and size between ε^∆C^ mutant and parental strain was detected (data not shown). Two hours after inoculation (exponential phase), the concentration of ATP in the ∆*rrn8* ε^∆C ^cells was significantly higher than that in the ∆*rrn8* ε^wt^; however, at 4 hr after inoculation, the ATP concentration in the ∆*rrn8* ε^∆C ^cells, but not in the ∆*rrn8* ε^wt^ cells, was significantly decreased (Figure 4b). On the other hand, the membrane potentials of both ∆*rrn8* ε^∆C ^mutant cells and ∆*rrn8* ε^wt ^cells were decreased at 4 hr after inoculation (Figure 4c). The observed decrease in the cellular membrane potential of the ∆*rrn8* ε^∆C ^mutant was, however, higher than that of ∆*rrn8* ε^wt^, and the recovery of the membrane potential at 5 hr after inoculation in ∆*rrn8* ε^∆C ^cells was quite poor compared to that in ∆*rrn8* ε^wt^ cells (Figure 4c). Therefore, it is likely that the observed decrease in the cellular membrane potential of ∆*rrn8* ε^∆C ^was probably due to the reduction in the H^+^‐pumping activity of BF_o_F_1_.

### 3.4 A search for a factor which triggers growth stalling in ∆*rrn8* ε^∆C ^mutant cells

**Figure 5:**
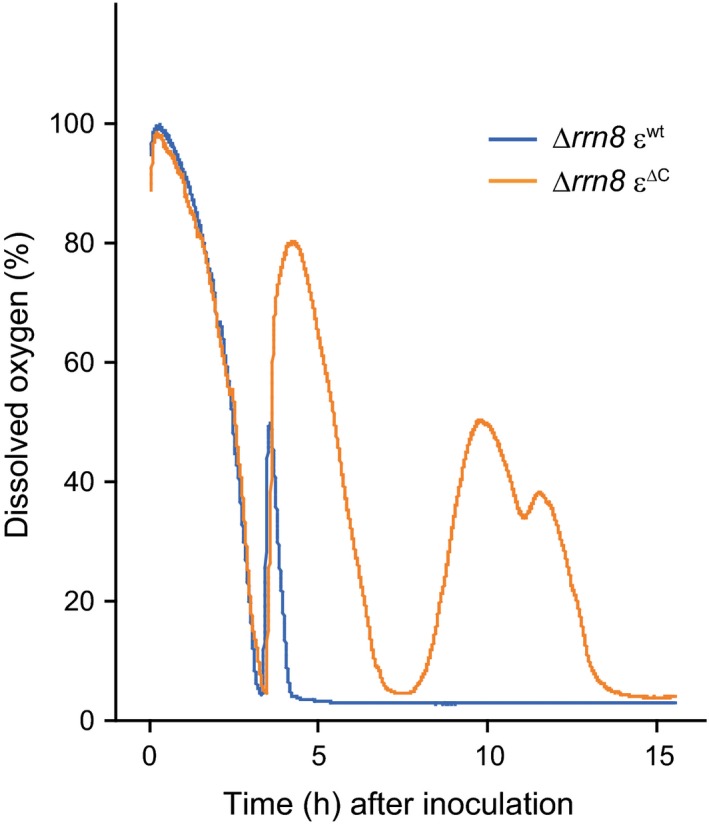
Time dependent changes in the concentration of dissolved oxygen in the cell culture. ∆*rrn8* ε^wt^ (blue line) and ∆*rrn8* ε^∆C^ (orange line) cells were cultured at 37°C with shaking in LB medium. The dissolved oxygen concentration was directly measured using a FIBOX3 oxygen meter. Results shown are means of three independent experiments

**Figure 6:**
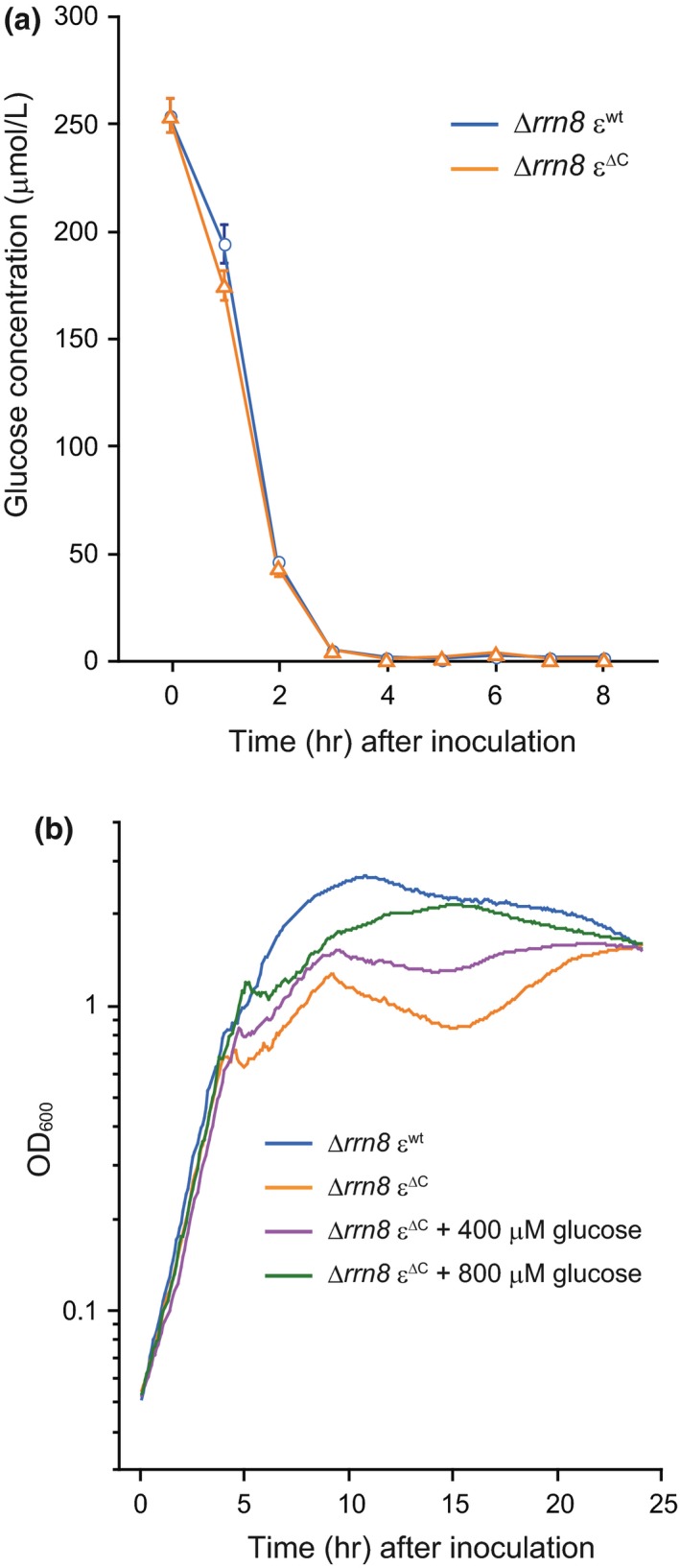
Relationship between the stalling of growth of ∆*rrn8* ε^∆C^ cells and glucose concentration in the culture. (a) Time dependent changes in the glucose concentration in the cultures of ∆*rrn8* ε^wt^ (blue circles) and ∆*rrn8* ε^∆C^ (orange triangles) cells, which were grown in LB medium. The glucose concentration in the culture supernatant of cells harvested at the indicated times was measured as described in the Materials and Methods. Results shown are averages of three independent experiments. Error bars indicate standard errors. (b) The effect of addition of glucose on the growth of the ∆*rrn8* ε^∆C^ mutant. Growth curves of ∆*rrn8* ε^wt^ and ∆*rrn8* ε^∆C^ cells grown in LB medium without any added glucose are shown using blue line and orange line, respectively. Growth curves of ∆*rrn8* ε^∆C^ mutant cells in LB medium supplemented with either 400 µmol/L (purple line) or 800 µmol/L (green line) glucose. OD_660_ nm of aliquots taken from the culture was measured every 5 min

**Figure A4:**
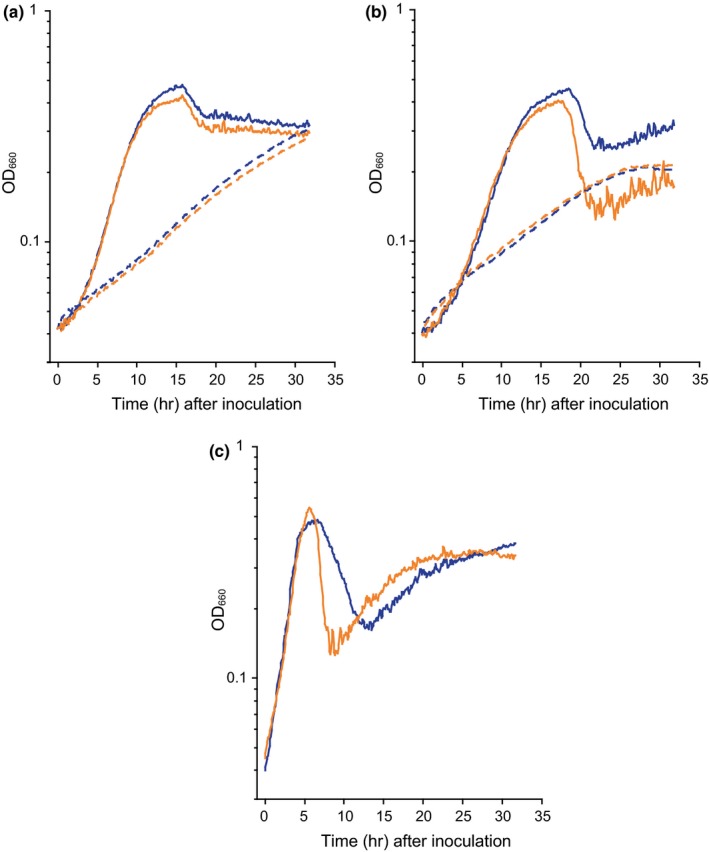
Growth curves of each mutant in minimal medium. In (a) and (b), cells were grown in minimal medium with glucose (solid lines) or succinate (dashed lines). (a) Growth curves of WT ε^wt^ (blue lines) and WT ε^∆C^ mutant (orange lines). (b) Growth curves of ∆*rrn8* ε^wt^ (blue lines) and ∆*rrn8* ε^∆C^ mutants (orange lines). In (c), ∆*rrn8* ε^wt^ (blue line) and ∆*rrn8* ε^∆C^ mutants (orange line) were grown in minimal medium containing both glucose and casamino acid. OD_660_ value of each culture was measured every 5 min

As shown above, introduction of the ε^∆C ^mutation into the ∆*rrn8 *strain enhanced the effect of C‐terminal domain deletion of the ε subunit and caused an obvious growth phenotype. Therefore, we expected that elucidation of the underlying mechanism of growth stalling observed in the case of the ∆*rrn8* ε^∆C ^mutant might provide some insight into the physiological significance of the regulation of BF_o_F_1_ by the ε subunit. During the growth‐stalling phase of the ∆*rrn8* ε^∆C ^mutant, a reduction in the membrane potential, which most likely correlates with the decrease in the PMF, was also observed (Figure 4c). The PMF of *B. subtilis* grown under aerobic conditions is mainly generated by the respiratory complex that consumes molecular oxygen as the final electron acceptor (Azarkina et al., 1999; García Montes de Oca et al., 2012; Lauraeus & Wikström, 1993; Winstedt & von Wachenfeldt, 2000), and thus the activity of the respiratory complex was monitored by measuring the dissolved oxygen concentration in the culture medium. When the respiratory complex activity is high, dissolved oxygen in the culture is continuously consumed, whereas a decrease in the activity of the respiratory complex allows the concentration of dissolved oxygen to increase by dissolution of oxygen into the culture from air (Shimada & Tanaka, 2016). Figure 5 shows the growth‐dependent changes in the dissolved oxygen levels in the culture of each strain. In both cultures, the levels of dissolved oxygen continuously decreased until approximately 3.5 hr after inoculation, and then consumption of oxygen was transiently arrested, following which the levels of dissolved oxygen in both cultures increased. In the case of ∆*rrn8* ε^wt ^strain, the dissolved oxygen level increased to 50%, and then oxygen consumption was suddenly resumed (Figure 5). In contrast, the dissolved oxygen level in the culture of ∆*rrn8* ε^∆C ^mutant cells increased to about 80% before oxygen consumption was resumed, thus delaying the reinitiation of oxygen consumption by the ∆*rrn8* ε^∆C ^mutant as compared to that by the ∆*rrn8* ε^wt ^strain. These results suggest that the activity of the respiratory complex in both strains was arrested 3.5 hr after inoculation, and the arrest of activity of the respiratory complex was extended in the ∆*rrn8* ε^∆C ^mutant. Succinate and NADH, which are driving forces of the respiratory complex (Azarkina et al., 1999; García Montes de Oca et al., 2012; Lauraeus & Wikström, 1993; Winstedt & von Wachenfeldt, 2000), are synthesized via glycolysis and the TCA cycle. Because glucose is the starting substrate for glycolysis and is metabolized via the TCA cycle, we measured the amount glucose remaining in the culture. Before cultivation, LB medium contained approximately 250 µmol/L of glucose, which was derived from the yeast extract and/or tryptone. However, the amount of glucose remaining in the cultures of the ∆*rrn8* ε^∆C ^mutant and ∆*rrn8* ε^wt ^strain by 3 hr after inoculation was 5.8 µmol/L and 5.3 µmol/L, respectively, and at 4 hr after inoculation was 1.8 µmol/L and 1.7 µmol/L, respectively (Figure 6a), suggesting that the glucose in the culture medium was mostly utilized by 3 hr after inoculation and depleted at 4 hr after inoculation. Because the time at which the growth of the ∆*rrn8* ε^∆C ^cells began to stall was same as the time at which glucose was depleted from the culture medium, we next examined the effect of addition of glucose to the culture on the growth of the ∆*rrn8* ε^∆C ^mutant cells. When glucose was added to the culture of the ∆*rrn8* ε^∆C ^mutant before starting cultivation, growth stalling was delayed (Figure 6b). These results suggested that the depletion of glucose in the culture medium is one of the factors that triggered the stalling of growth of the ∆*rrn8* ε^∆C ^mutant cells. This hypothesis is partly supported by the competitive survival disadvantage of the WT ε^∆C^ mutant when co‐cultured with the wild‐type cells, which was suppressed by the addition of glucose to the culture (Figure 3b). Accordingly, when the WT ε^∆C^ mutant cells were co‐cultured with the wild‐type cells in LB medium, the relative abundance of WT ε^∆C^ mutant cells was <20% of its original abundance after three subcultures; in contrast, when the WT ε^∆C^ mutant cells grown with the wild‐type cells in LB medium that was supplemented with 0.1% glucose, the relative abundance of these cells virtually remained unchanged (Figure 3b). To identify the factor that triggers the growth stalling in the ∆*rrn8* ε^∆C ^mutant, we tried to observe the growth stalling in the defined medium. When WT ε^∆C^ mutant and ∆*rrn8* ε^∆C ^mutant cells were inoculated into the minimal medium containing glucose (MM glucose) or succinate, the growth rates of these mutants were the same as those of parental strains (Figure A4). We further added casamino acid to the MM glucose and observed the growth of the ∆*rrn8* ε^∆C ^mutant cells in this medium. However, the growth stalling of the ∆*rrn8* ε^∆C ^mutant cells observed in the LB medium was not observed in these minimal media (Figure A4c). Nevertheless, the decrease in the OD value after stopping the growth in the MM glucose and MM glucose with casamino acid, probably caused by cell lysis, of the ∆*rrn8* ε^∆C ^mutant cells occurs more severely than that of the ∆*rrn8* ε^wt^ cells (Figure A4b,c). This result implies, at least in part, the importance of the regulation of BF_o_F_1_ by ε subunit when glucose is depleted.

### 3.5 Reduction of cellular membrane potential induces decrease in the amount of 70S ribosomes

**Figure 7:**
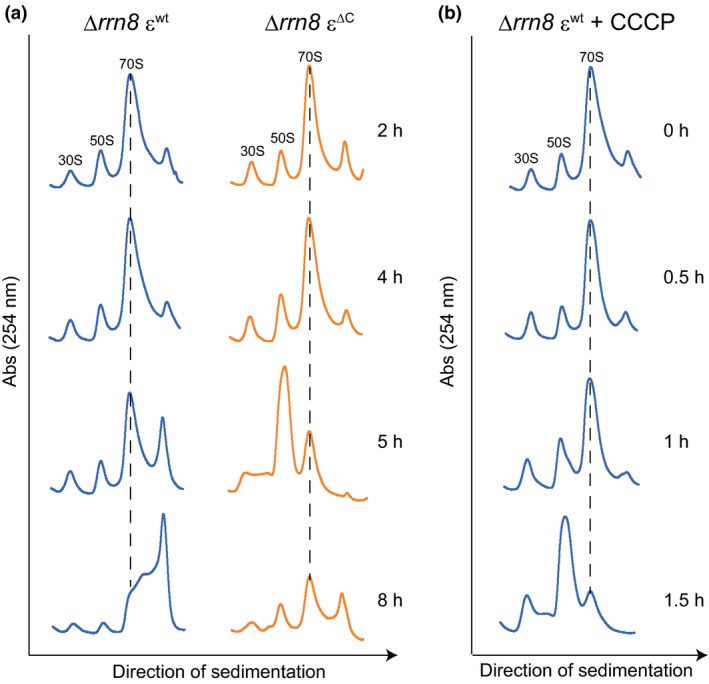
Ribosome sedimentation profiles of ∆*rrn8* ε^wt^ (blue lines) and ∆*rrn8* ε^∆C^ (orange lines) mutants. (a) Crude cell extracts, prepared from cells grown in LB medium and harvested at the indicated times, were subjected to sucrose density‐gradient centrifugation. (b) Effect of addition of uncoupler on the ribosome profile of ∆*rrn8* ε^wt^. Cells were grown in LB medium. At 4 hr after inoculation, 100 µmol/L of CCCP was added to the culture. Cells were harvested at the indicated times after the addition of CCCP. Crude cell extracts prepared from these cells were subjected to sucrose density‐gradient centrifugation. The 30S, 50S subunits and 70S ribosome peaks are indicated at the top of each profile. Dotted lines in each profile indicate the 70S ribosome peaks

We next examined why cell proliferation was stalled. Taking into consideration that growth stalling was observed in cells with ∆*rrn8* genetic background (i.e., in cells with decreased amount of ribosomes), we examined the ribosome profiles of the ∆*rrn8* ε^∆C ^mutant cells at different stages of the growth cycle. For this purpose, cells were grown in LB and the ribosome profiles of cells at different stages of growth were analyzed by using a sucrose density gradient sedimentation assay. Surprisingly, we observed a decrease in the amount of 70S ribosomes and an aberrant accumulation of 50S subunits in the ∆*rrn8* ε^∆C ^mutant cells 5 hr after inoculation, which corresponded to the growth‐stalling phase (Figure 7a). Although a modest decrease in the 70S peak was also detected in the ∆*rrn8* ε^wt^ cells 5 hr after inoculation, the aberrant accumulation of subunits was not observed; in addition, the peak height of an additional peak was increased (Figure 7a). This additional peak probably contained polysomes, which consist of active 70S ribosomes bound to mRNAs, as well as ribosome‐dimers (100S ribosomes), because the growth phase of these ∆*rrn8* ε^wt^ cells corresponded to early stationary‐phase. We previously reported that ribosome‐dimers are formed at early stationary‐phase in *B*. *subtilis *(Akanuma et al., 2016). Because the bacterial ribosome forms a translationally active 70S complex by assembling 30S and 50S subunits, the most likely reason for the stalling of growth of ∆*rrn8* ε^∆C ^mutant cells is the decrease in the amount of 70S ribosomes, and the accompanying reduction in the translational activity of the cell. However, an important question that remained to be answered was why the amount of 70S ribosomes decreased? The observed reduction in the membrane potential of ∆*rrn8* ε^∆C ^mutant cells during growth stalling (Figure 4c) predicted that the decrease in the PMF might be responsible for reducing the amount of 70S ribosome. To test this hypothesis, CCCP was added to the culture of ∆*rrn8* ε^wt^ cells 4 hr after inoculation, and the culture was grown without interruption. We have already observed a decrease in the membrane potential of ∆*rrn8* ε^wt^ cells following the CCCP treatment (Figure 4c). One hour after the addition of CCCP to the culture of ∆*rrn8* ε^wt^ cells, the height of the 70S peak slightly decreased with concomitant increases in the heights of 30S and 50S subunit peaks; moreover, 1.5 hr after the addition of CCCP, a significant decrease in the 70S ribosome peak with an aberrant accumulation of 50S subunits was observed (Figure 7b). These results suggest that the decrease in the amount of 70S ribosome during the growth stalling period of the ∆*rrn8* ε^∆C ^mutant was triggered by the reduction in the PMF.

## 4 DISCUSSION

In the present study, we presented biochemical evidence that CTD, the regulatory region of the ε subunit, activates the H^+^ pumping activity of BF_o_F_1_, probably by enhancing its ATP hydrolysis activity. Moreover, phenotypic analysis of WT ε^∆C^ and ∆*rrn8* ε^∆C^ mutant cells showed that the CTD of the ε subunit plays an important role in the maintenance of cellular membrane potential.

### 4.1 ε Subunit‐mediated regulation of BF_o_F_1 _is different than that in other species

The ε^∆C^ mutation slightly decreased ATP‐synthesis activity of BF_o_F_1_, although this seems to be in parallel with the decreased H^+^‐pumping activity of the respiratory complex in the ε^∆C^ mutant cells (Figure 2a,c). In contrast to what we have observed here, truncation of the CTD of the ε subunit both in TF_o_F_1_ and EF_o_F_1 _led to an increase in the ATP synthesis activity of F_o_F_1_ by relieving the repression that was caused by the ε subunit (Iino, Hasegawa, Tabata, & Noji, 2009; Masaike, Suzuki, Tsunoda, Konno, & Yoshida, 2006; Taniguchi et al., 2011). In these previous studies, truncation site of the CTD of the ε subunit of TF_o_F_1_ was at residues V90, and that of the ε subunits of EF_o_F_1 _were at residues D91 or R94. In the present case, the truncation site of the CTD of the ε^∆C ^mutant was at residue K90. According to the amino acid sequence alignment analysis of the ε subunits of BF_o_F_1_, TF_o_F_1_ and EF_o_F_1_, the K90 in the ε subunit of BF_o_F_1_ corresponded to the V90 of TF_o_F_1_ and E92 of EF_o_F_1_, all of which were closely located. Thus, it is unlikely that the observed difference in the effect of ε^∆C ^mutation is due to the truncation length of the CTD; instead it suggests diversity of the mechanism by which the ε subunit regulates ATP synthesis.

The role of ε subunit in BF_1_‐ATPase is different from its role in other well‐known F_1_‐ATPases. In BF_1_, the ε subunit activates the ATPase activity (Mizumoto et al., 2013), whereas in other F_1_‐ATPases, the ε subunit generally inhibits the ATPase activity (Kato et al., 1997; Laget & Smith, 1979; Ort & Oxborough, 1992; Smith et al., 1975). Results obtained from this study demonstrate that even in the BF_o_F_1_ complex, the regulatory role of the ε subunit is different from that in the other F_o_F_1_s. It has been reported that the ATPase and H^+^‐pumping activities of TF_o_F_1_, EF_o_F_1_, and the F_o_F_1 _of thermoalkaliphile *Bacillus* sp. strain TA2.A1 (TAF_o_F_1_), containing the C‐terminal truncated ε subunit (TF_o_F_1_; deleted from residues 90 to 133, EF_o_F_1_; deleted from residue 94 to 139 or 91 to 139, TAF_o_F_1_; deleted from residue 90 to 134. See Figure 1) were relatively higher than those of the intact F_o_F_1_s (Iino et al., 2009; Kato‐Yamada et al., 1999; Keis et al., 2006; Taniguchi et al., 2011), results that are in contrast to what we observed here for the BF_o_F_1 _(Figure 2b,c). As described above, the roles of ε subunit are diverse. In BF_o_F_1_ the ε subunit most likely plays a key role in the activation of ATPase and H^+^‐pumping activity, because the ε^∆C^ mutation affected the ATP hydrolysis activity rather than the ATP synthesis activity. ATP synthesis reaction need not be strictly controlled because it always requires PMF. On the other hand, ATP hydrolysis reaction that may lead waste of ATP should be controlled strictly, as it can occur under any situation with lowered PMF. The asymmetric effect of the mutation on ATP synthesis and hydrolysis observed here may relate to such requirement to this enzyme.

However, the detail of the mechanism for how C‐terminal domain of the ε subunit activates ATP hydrolysis activity of BF_o_F_1_ remains unclear, because the ATP hydrolysis activity in the IMV of *B. subtilis* is not high enough to solve this question. There is a possibility that C‐terminal domain of ε subunit actively enhances ATP hydrolysis of BF_o_F_1_ by removing the MgADP inhibition. Concomitantly, the entire ε subunit might be prerequisite for maximum activity of ATP hydrolysis of BF_o_F_1_, like the F_o_F_1 _of *Mycobacterium tuberculosis *(Bogdanović et al., 2018). The detailed mechanism for the activation of ATP hydrolysis activity of BF_o_F_1_ by the ε subunit will be revealed by analysis using IMV containing abundant BF_o_F_1_ that are artificially overexpressed.

### 4.2 Possible physiological significance of the ε subunit‐mediated regulation of BF_o_F_1_

What is the physiological significance of the regulation of the BF_o_F_1_ mediated by the ε subunit? Based on our data, we suggest that the ε subunit plays an important role in alleviating the temporal reduction in the membrane potential by enhancing the H^+^ pumping activity of F_o_F_1_. The membrane potential of the ∆*rrn8* ε^∆C^ mutant was found to be comparatively less than that of the ∆*rrn8* ε^wt^ strain, particularly at the growth‐stalling phase (Figure 4c). Given that the decrease in the membrane potential was caused by the reduction in the PMF, the defect in the H^+^ pumping activity of F_o_F_1 _probably contributed to the stalling of growth of the ∆*rrn8* ε^∆C ^mutant. A decrease in the PMF affects many cellular functions, such as translocation of amino acids and proteins (Schulze et al., 2014; Tolner, Ubbink‐Kok, Poolman, & Konings, 1995). In addition, the PMF is known to modulate the distribution of several conserved cell division proteins such as MinD, FtsA, and also the bacterial cytoskeletal protein MreB (Strahl & Hamoen, 2010). Thus, the decrease in PMF may adversely affect all or some of these cellular functions and may thus promptly inhibit cell proliferation, leading to stalling of growth of the ∆*rrn8* ε^∆C^ mutant. A decrease in the amount of 70S ribosomes in the ∆*rrn8* ε^∆C ^mutant, which would reduce the translational activity of these cells, would probably extend the growth stalling period. Our observation that the addition of CCCP to the culture of the ∆*rrn8* ε^wt^ cells led to an increase in the amount of 30S and 50S subunits with a concomitant decrease in the amount of 70S ribosomes (Figure 7b) suggested that a reduction in the PMF triggered the dissociation of 70S ribosomes into 30S and 50S subunits. Although the mechanism by which a reduction in the PMF leads to the dissociation of the 70S ribosomes to 30S and 50S subunits in the ∆*rrn8* cells remains unclear, the addition of CCCP to the wild‐type cells led to the formation of ribosome dimers (result not shown). Ribosome dimers are a translationally inactive form of ribosomes that are formed in the stationary phase (Akanuma et al., 2016; Yoshida et al., 2002). Therefore, *B*. *subtilis *apparently tries to suppress the translational activity when the PMF of the cell is decreased.

### 4.3 Circumstances requiring H^+ ^pumping by BF_o_F_1_

Although the difference in the membrane potential between the ∆*rrn8* ε^wt^ and ∆*rrn8* ε^∆C ^cells was caused by the decrease in the H^+^ pumping activity of F_o_F_1_, the primary reduction in the membrane potential, which was observed in both ∆*rrn8* ε^wt^ and ∆*rrn8* ε^∆C ^cells at 4 hr after inoculation, was probably caused by the arrest of H^+ ^pumping activity of the respiratory complex. Consequently, the consumption of dissolved oxygen by cells in the culture, an indicator of the activity of the respiratory complex, arrested just before the decrease in the membrane potential in both ∆*rrn8* ε^wt^ and ∆*rrn8* ε^∆C ^cells, although the arrest of respiration was temporary in the ∆*rrn8* ε^wt^ strain (Figures 4c and 5). There is a possibility that the arrest of the H^+ ^pumping activity of the respiratory complex was induced by the depletion of glucose in the culture medium, because glucose, which is the usual substrate for glycolysis and the TCA cycle and drives the respiratory complex, was depleted synchronously with the arrest of dissolved oxygen consumption (Figure 6a). Given that the temporary decrease in the membrane potential was triggered by glucose depletion, the activation of the H^+^ pumping activity of BF_o_F_1_, mediated by the ε subunit, is required to counteract the reduction in the PMF caused by the depletion of glucose. When glucose is depleted, the amount of 70S ribosomes would remain unchanged if the membrane potential could be recovered immediately by increasing the H^+^ pumping activity of F_o_F_1_. The cellular translational activity may be maintained by allowing the cells to synthesize the proteins that are required for the utilization of carbon sources other than glucose, such as importers of other sugars and their metabolic enzymes, importers of TCA‐cycle intermediates, and metabolic enzymes for fatty acid catabolism (Fujita, 2009). Our observation that glucose supplementation to the culture medium suppressed the observed decrease in the survival rate of the WT ε^∆C ^mutant under the competitive condition (Figure 3b), partly supports this hypothesis. The effect of ε^∆C ^mutation on the growth of the wild‐type was less than that of the ∆*rrn8* mutant. It is possible that the presence of sufficient amount of ribosomes in the wild type cells may mask these defects by maintaining the cellular translational activity.

### 4.4 Contribution of the conformational change in the ε subunit to the regulation of BF_o_F_1_

**Figure 8:**
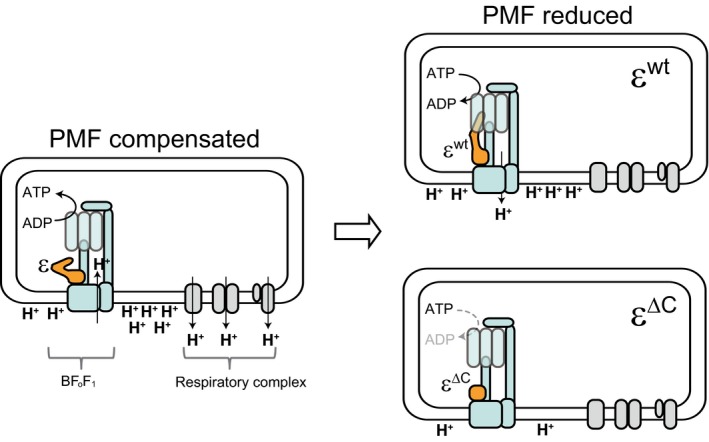
The possible role of ε subunit in *Bacillus subtilis*. When there is enough PMF across the cell membrane, the BF_o_F_1_ mainly synthesize ATP using the PMF (left). On the other hand, when the PMF is decreased, for example which is caused by reduction in the activity of respiratory complex after depletion of glucose, the ε subunit activates the H^+^ pumping activity of BF_o_F_1_ by stimulating its ATP hydrolysis activity to alleviate the reduction of PMF (right, top). If the BF_o_F_1_ is unable to export sufficient amount of H^+^ due to the ε^∆C^ mutation, the PMF is continuously decreased (right, bottom). As a result, some of the important cellular functions, such as reduction in the translational activity, are adversely affected

Previous studies have suggested a correlation between the conformational change in the CTD of the ε subunit and its regulatory function (Feniouk et al., 2010; Iino et al., 2005; Kato et al., 2007; Rodgers & Wilce, 2000; Tsunoda et al., 2001). In general, when the CTD of the ε subunit adopts an extended conformation, it inhibits ATPase activity; whereas in the folded‐state conformation, the CTD of the ε subunit does not inhibit ATPase activity. However in BF_o_F_1_, the extended conformation of the ε subunit probably activates the ATPase and H^+^ pumping activities, because we have reported earlier that the extended conformation of the ε subunit activates the ATP hydrolysis activity of BF_1_ (Mizumoto et al., 2013). Consistent with this idea, in the present study we showed that the deletion of CTD of the ε subunit, which mimics the folded‐state, decreased the ATPase and H^+^ pumping activities. The ATP concentration affects the conformational state of the ε subunit in TF_o_F_1_ (Kato et al., 2007), because the folded‐state of ε subunit is stabilized by binding of ATP to the CTD (Iino et al., 2005; Yagi et al., 2007). We have also demonstrated that an ATP molecule binds to the ε subunit of BF_o_F_1_ (Kato‐Yamada, 2005). Therefore, in *B*. *subtilis *cells, the CTD of the ε subunit may change its conformation by sensing the cellular ATP concentration to regulate the activity of BF_o_F_1_. Given that the reduction in the PMF triggers a decrease in the cellular ATP concentration, which is caused by a decrease in the activity of oxidative phosphorylation, the ε subunit can sense the reduction in the PMF via the decrease in the cellular ATP concentration, and in response activates the H^+^ pumping activity of BF_o_F_1_. Although at present we cannot fully explain the relationship between the conformational change in the CTD of the ε subunit and its regulatory function, we have proposed a model (Figure 8) suggesting the possible role of the ε subunit in *B. subtilis*.

### 4.5 Concluding remarks

The ATPase activity of F_o_F_1 _must be tightly controlled because any waste of ATP, which is utilized by many cellular processes, would adversely affect cell survival, particularly in the natural environment. Accordingly, the inhibition of BF_1 _by MgADP is very tightly controlled to avoid wasting ATP (Mizumoto et al., 2013). However, in response to the reduction in the PMF, which adversely affects the cell function, BF_o_F_1 _generates the PMF using the chemical energy stored in the ATP. The CTD of the ε subunit, on the other hand, inhibits the ATPase activity of F_o_F_1_ *,* which is frequently subjected to anoxic conditions (Klionsky, Brusilow, & Simoni, 1984; Kuki et al., 1988). Moreover, the ε subunit inhibits ATP hydrolysis and product release in *E.coli*, and inhibits ATP binding in *Bacillus *PS3 (Iino et al., 2009; Kato et al., 1997; Yasuno, Muneyuki, Yoshida, & Kato‐Yamada, 2009). These findings demonstrate that the basic characteristics of F_o_F_1 _regulation differ considerably in different bacteria. Therefore, it is likely that the ε subunit in F_o_F_1_‐ATPase/synthases has evolved various regulatory functions to allow diverse bacteria to adapt to different habitats. Because of the variety of the regulatory mechanism by the ε subunit, it has also been attracted considerable attention as a potential drug target (Biukovic et al., 2013; Joon et al., 2018). The elucidation of the physiological significance of the regulation of the F_o_F_1_‐ATPase/synthases in various species, such as the γ subunit‐mediated regulation of the cyanobacterial F_o_F_1_ maintain the intracellular ATP level (Sunamura, Konno, Imashimizu‐Kobayashi, Sugano, & Hisabori, 2010), will reveal the relationship between the evolution of the regulatory system for F_o_F_1_ and the environment in which the organism lives.

## CONFLICT OF INTERESTS

The authors confirm that this article content has no conflict of interest.

## AUTHORS CONTRIBUTION

YKY, GA and FK performed the conception and design of the study; GA, TT, MS, SS, and TS performed the experiments; GA, TT, TS and KT performed analysis or interpretation of the data; GA and YKY wrote the manuscript.

## ETHICS STATEMENT

None required.

## Data Availability

All data is provided in full in the results section of this paper.

## APPENDIX 1

**Table A1:** Primers used in this study

Primer	Sequence (5’ to 3’)
atpC+catUF	GACAGGCCTTACAATGGCTG
atpC+catUR	TGATAATAAGGGTAACTATTGCCGGGAGAAGGATTTTTTCTTATTTCCC
atpC+catDF	AGGCCTAATGACTGGCTTTTATAAGAAAAAATCCTTCTCTTTATGAGAA
atpC+catDR	CCGTTCCAATGGCGATTGTC
atpC+catCF	GGCAATAGTTACCCTTATTATCAAGATA
atpC+catCR	TTATAAAAGCCAGTCATTAGGC
atpC∆CcatR	GGGAGAAGGATTTTTTCTTAATCGATGCCTTCCGCTGTCT
atpC∆CcatF	AGACAGCGGAAGGCATCGATTAAGAAAAAATCCTTCTCCC
atpCtfF	AATGGGTATTTACCCTGCGG
atpCtfR	ATATTGACGGCTTGCAACGC
pDGinfF	GCTAATTCGGTGGAAACGAGGTCATC
pDGinfR	GCATGCCTGCAGGTCGACTCTAGAT
pDGatpCF	GACCTGCAGGCATGCAAGTTTAATCTGGTCCTAGG
pDGatpCR	TTCCACCGAATTAGCCCGTTGCTGTCGCTGTTT
